# Supplementary material for: Integrated lipidomic and transcriptomic analysis reveals diacylglycerol accumulation in olive of Longnan (China)
Source: PeerJ. 2023 Aug 11;11:e15724. doi: 10.7717/peerj.15724 (PMC10424668; doi:10.7717/peerj.15724)
Supplement: Supplemental Information 1 — The data are shown as means of three biology repeats ± SD. [file peerj-11-15724-s001.docx]

**
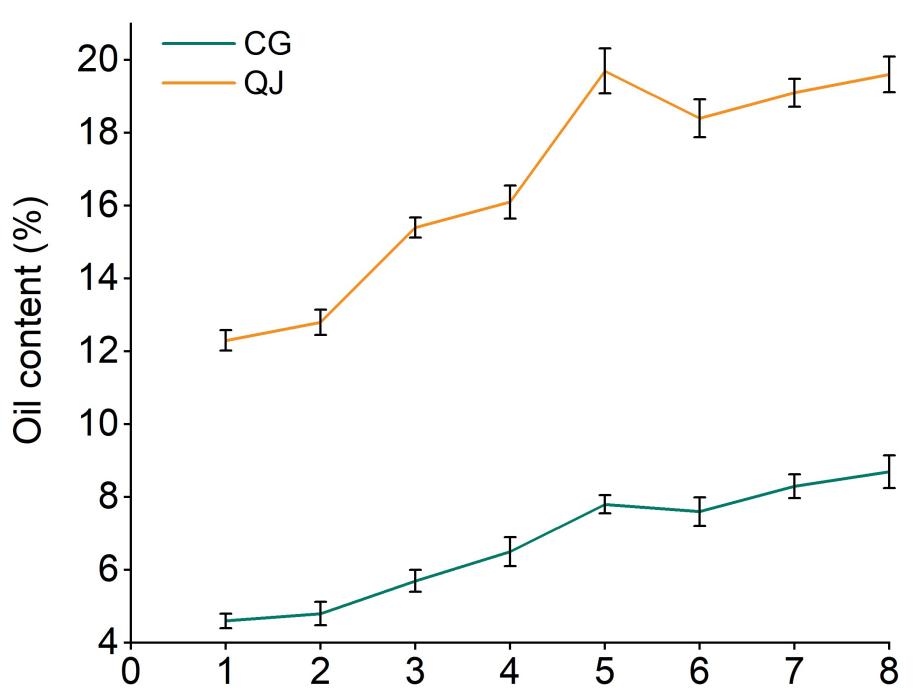
**

**Supplementary Figure 1.** Oil content of fresh olive fruit at different maturity stages. The data are shown as means of three biology repeats±SD.
