# Supplementary material for: Integrated lipidomic and transcriptomic analysis reveals diacylglycerol accumulation in olive of Longnan (China)
Source: PeerJ. 2023 Aug 11;11:e15724. doi: 10.7717/peerj.15724 (PMC10424668; doi:10.7717/peerj.15724)
Supplement: Supplemental Information 2 — The different lipid class are represented by different colours and the proportion of them is indicated by the size of the colour block area. [file peerj-11-15724-s002.docx]

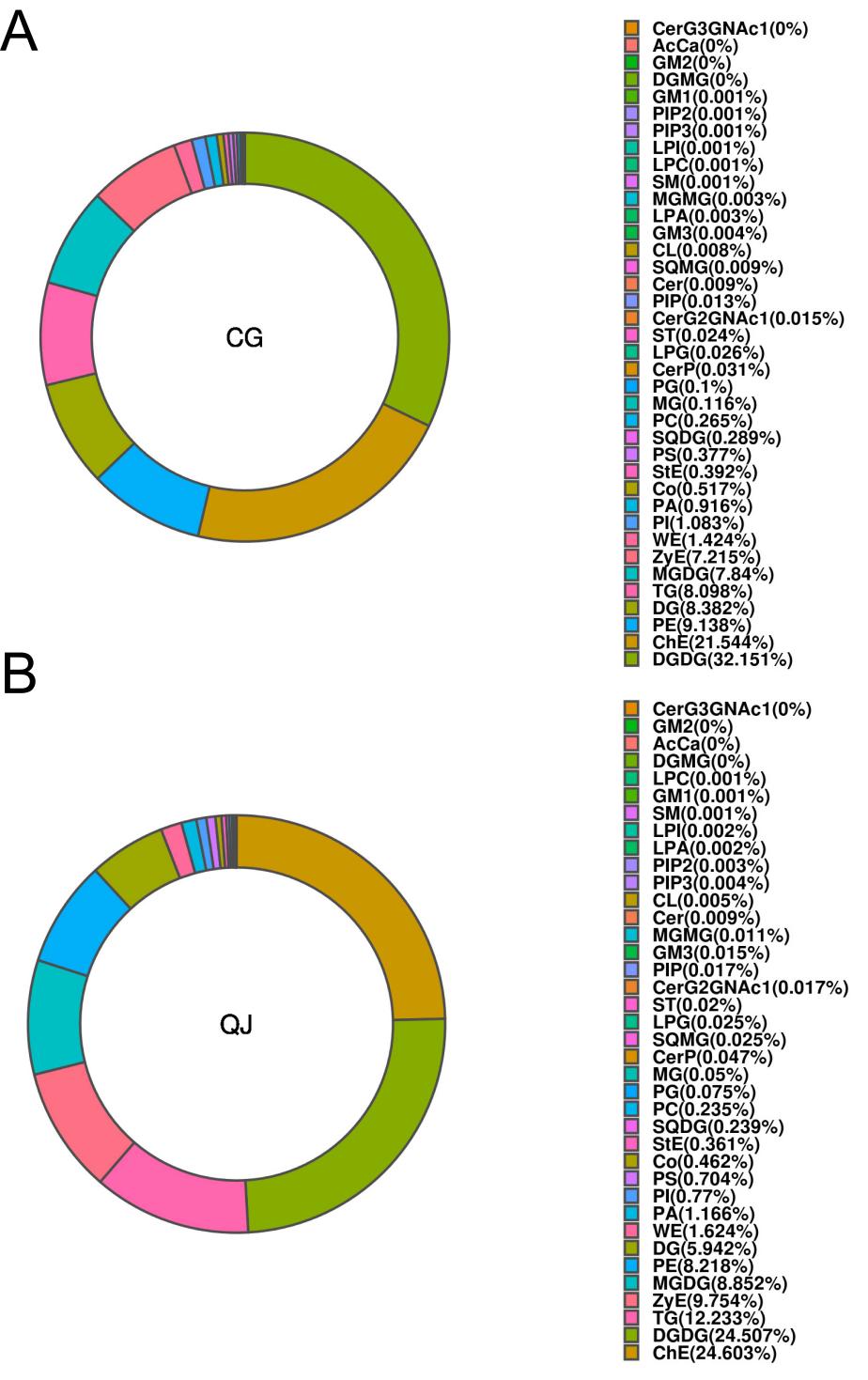


**Supplementary Figure 2.** Composition of lipid class (CG vs. QJ). The different lipid class are represented by different colours and the proportion of them is indicated by the size of the colour block area.
