# Supplementary material for: Integrated lipidomic and transcriptomic analysis reveals diacylglycerol accumulation in olive of Longnan (China)
Source: PeerJ. 2023 Aug 11;11:e15724. doi: 10.7717/peerj.15724 (PMC10424668; doi:10.7717/peerj.15724)
Supplement: Supplemental Information 3 — In the figure t[1] represents principal component one, to[1] represents principal component two, and the ellipse represents the 95% confidence interval. Points of the same colour indicate individual biological replicates within the group, and the distribution status of the points reflects the degree of variation between and within groups.(R2X (cum):0.839, R2Y (cum):0.994). [file peerj-11-15724-s003.docx]

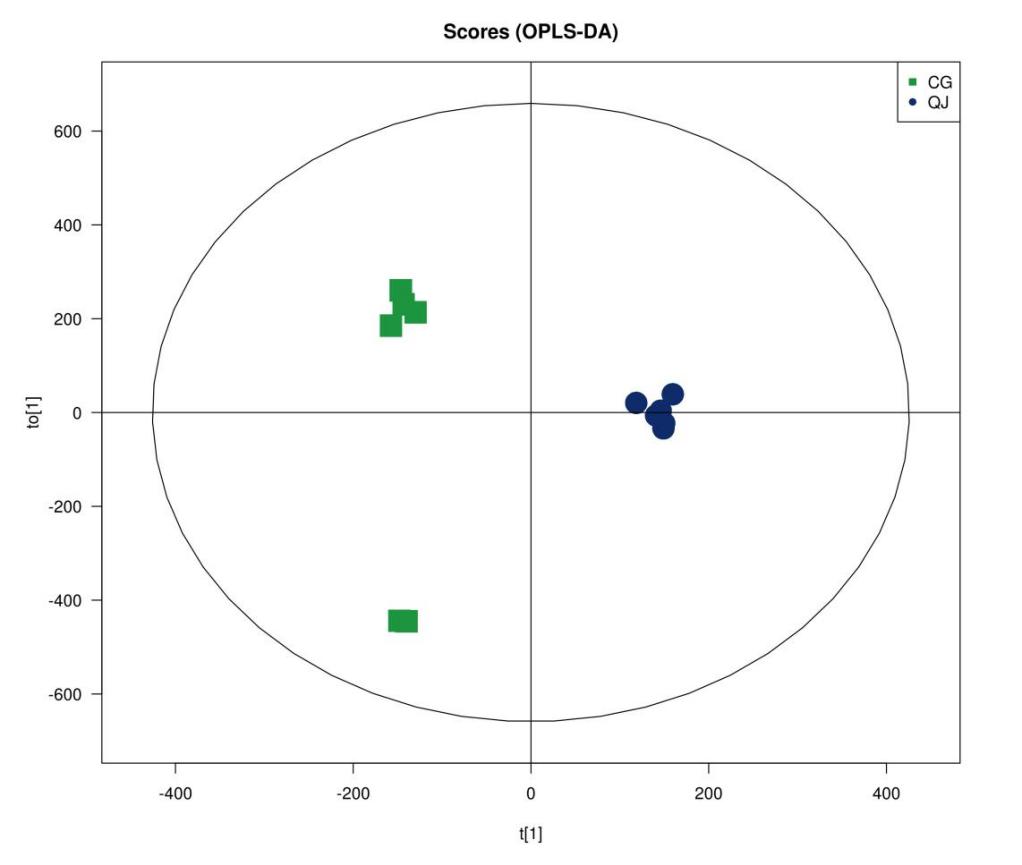


**Supplementary Figure 3.** Orthogonal partial least squares discriminant analysis (OPLS-DA) of lipidomic. In the figure t[1] represents principal component one, to[1] represents principal component two, and the ellipse represents the 95% confidence interval. Points of the same colour indicate individual biological replicates within the group, and the distribution status of the points reflects the degree of variation between and within groups.(R2X (cum):0.839, R2Y (cum):0.994).
