# Supplementary material for: Integrated lipidomic and transcriptomic analysis reveals diacylglycerol accumulation in olive of Longnan (China)
Source: PeerJ. 2023 Aug 11;11:e15724. doi: 10.7717/peerj.15724 (PMC10424668; doi:10.7717/peerj.15724)
Supplement: Supplemental Information 4 — The horizontal and vertical coordinates in the graph represent the samples and the numbers in the circles represent the correlation coefficients of the two samples. (A) Hierarchical cluster analysis (HCA) analysis was used to determine the variation in differential genes between groups. Color scale represents differential gene expression. Red color represents highly expressed gene and blue color represents low expressed gene (B). [file peerj-11-15724-s004.docx]

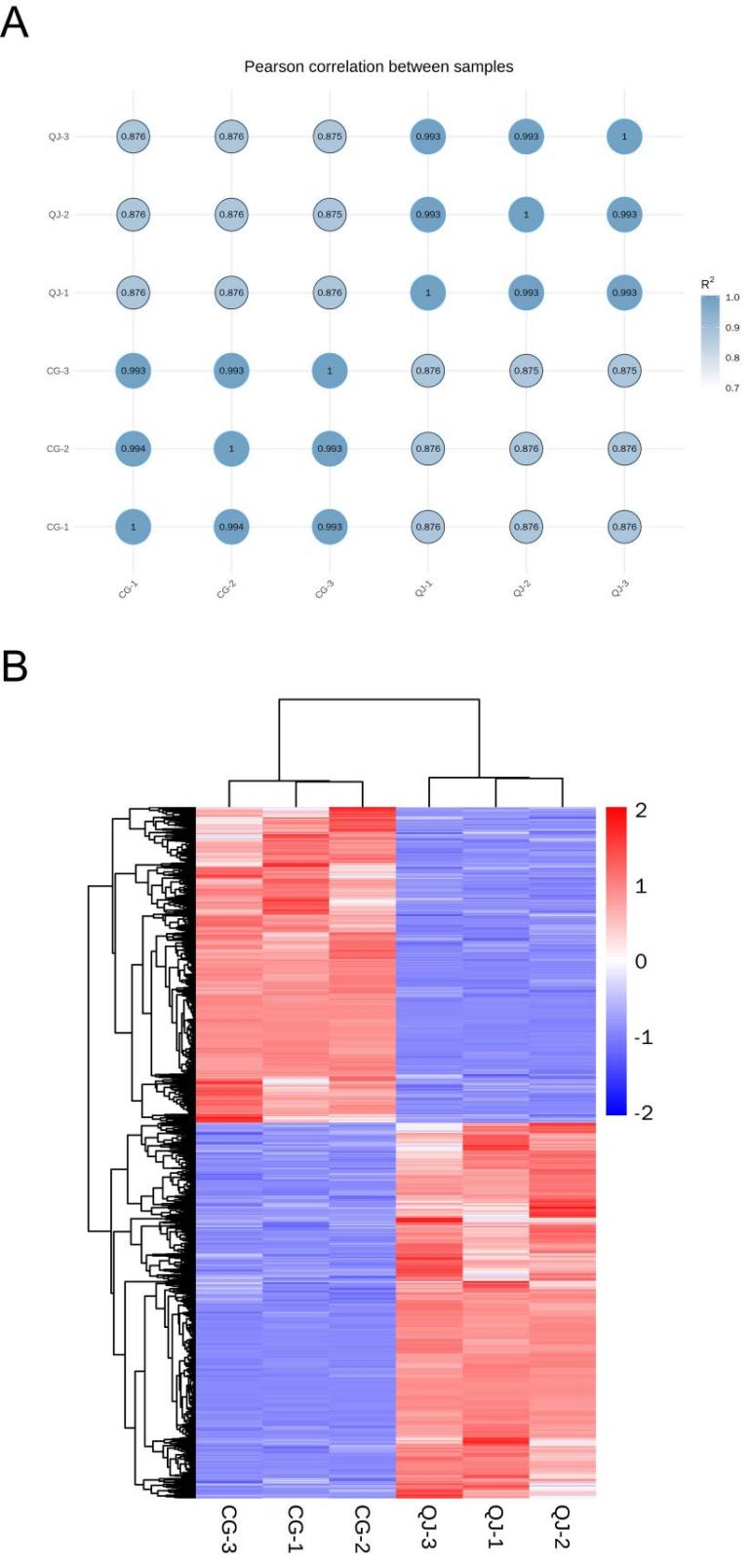


**Supplementary Figure 4.** Pearson correlation analysis between CG and QJ. The horizontal and vertical coordinates in the graph represent the samples and the numbers in the circles represent the correlation coefficients of the two samples. (A) Hierarchical cluster analysis (HCA) analysis was used to determine the variation in differential genes between groups. Color scale represents differential gene expression. Red color represents highly expressed gene and blue color represents low expressed gene.(B)
